# Chemokines in Alzheimer’s Disease: New Insights Into Prokineticins, Chemokine-Like Proteins

**DOI:** 10.3389/fphar.2019.00622

**Published:** 2019-05-29

**Authors:** Anna Rita Zuena, Paola Casolini, Roberta Lattanzi, Daniela Maftei

**Affiliations:** ^1^Department of Physiology and Pharmacology “Vittorio Erspamer,” Sapienza University of Rome, Rome, Italy; ^2^Department of Biochemical Sciences “Alessandro Rossi Fanelli,” Sapienza University of Rome, Rome, Italy

**Keywords:** Alzheimer’s disease, chemokines, prokineticin receptors, Aß-peptide, prokineticins

## Abstract

Alzheimer’s disease is the most common neurodegenerative disorder characterized by the presence of β-amyloid aggregates deposited as senile plaques and by the presence of neurofibrillary tangles of *tau* protein. To date, there is a broad consensus on the idea that neuroinflammation is one of the most important component in Alzheimer’s disease pathogenesis. Chemokines and their receptors, beside the well-known role in the immune system, are widely expressed in the nervous system, where they play a significant role in the neuroinflammatory processes. Prokineticins are a new family of chemokine-like molecules involved in numerous physiological and pathological processes including immunity, pain, inflammation, and neuroinflammation. Prokineticin 2 (PROK2) and its receptors PKR1 and PKR2 are widely expressed in the central nervous system in both neuronal and glial cells. In Alzheimer’s disease, PROK2 sustains the neuroinflammatory condition and contributes to neurotoxicity, since its expression is strongly upregulated by amyloid-β peptide and reversed by the PKR antagonist PC1. This review aims to summarize the current knowledge on the neurotoxic and/or neuroprotective function of chemokines in Alzheimer’s disease, focusing on the prokineticin system: it represents a new field of investigation that can stimulate the research of innovative pharmacotherapeutic strategies.

## Alzheimer’s Disease

Alzheimer’s disease (AD) is the most common progressive neurodegenerative disorder and is the most frequent cause of dementia, characterized by a progressive and irreversible mental decline with loss of cognitive skills and memory function. The histopathological hallmarks of AD are extracellular senile plaques that are aggregates of amyloid-β (Aβ) peptide, and intracellular aggregation of hyper-phosphorylated *tau* protein that forms neurofibrillary tangles (Haass and Selkoe, 2007; Huang and Mucke, 2012). This aggregation causes a neurotoxic cascade, which, in turn, leads to neuronal degeneration and atrophy of the brain regions involved in memory and cognitive impairment (temporal and parietal lobe, pre-frontal cortex, and hippocampus), increasing, in this way, brain neuroinflammation (Raskin et al., 2015; Bronzuoli et al., 2016). It is well known, in fact, that neuronal dysfunction is not the solely cause of AD pathogenesis and progression. There are increasing evidences showing that microglia and astrocytes are implicated in the neuroinflammatory reactions that characterize this pathology. Microglia cells are the innate immune cells of the central nervous system (CNS) and are involved in regulating synaptic plasticity and remodelling neuronal circuits. Astrocytes are the most numerous glial cells in the brain, and they provide nutrients and structural support to neurons. Moreover, microglia and astrocytes are responsible for brain homeostasis, and they react to disease stressors by innate immune responses such as production and release of inflammatory mediators that aim to resolve pathological state. In persistent pathological conditions, such as neurodegenerative diseases, however, microglia as well as astrocytes change their physiological phenotype and, consequently, lose their helpful function. Several studies from post-mortem brains of AD patients and AD animal models have revealed a co-localization of reactive glial cells with senile plaques and neurofibrillary tangles (Parachikova et al., 2007; Hickman et al., 2008; Lopez-Gonzalez et al., 2015). In particular, the early recruitment of microglia around plaques seems beneficial in AD by promoting phagocytosis of Aβ. However, the excessive amount of Aβ occurring with the disease progression overwhelms microglia, which loses its phagocytic capacity in favor of a pro-inflammatory role (Jay et al., 2015). It is known, in fact, that activation of microglia involves the release of several pro-inflammatory molecules (specifically IL-1β, TNFα and C1q) and induces the activation of astrocytes that consequently lose their neuroprotective activity (Liddelow et al., 2017). Astrocytes’ neurotoxic phenotype is abundant in AD patients’ brain. Therefore, in these conditions, microglia and astrocytes promote neuroinflammatory response, being responsible for the synthesis of different pro-inflammatory mediators including chemokines and mediators with chemokine-like function as defensins and macrophage migration inhibitory factor (MIF) (Casolini et al., 2002; Sudduth et al., 2013; Williams et al., 2013; Azizi et al., 2014; Guerriero et al., 2017; Chun et al., 2018).

This review aims to summarize the most current knowledge on role of chemokines in AD, focusing on the prokineticins, chemokine‐like molecules that have a role in the amyloid-induced neuronal damage (all the data shown below are summarized in **Table 1**).

**Table 1:** Summary of the effects of chemokines and prokineticins in different cellular and animal models of AD and their expression in AD patients.

Chemokines	Receptors	Effect on cellular or AD animal models	References	AD patients	References
CCL2 (MCP-1)	CCR2	CCR2 deficient mice: ↑ Aβ	El Khoury et al., 2007; Naert and Rivest, 2011	CCL2 ↑ in brain CCL2 ↑ in CSF CCL2 ↑ in plasma	Grammas and Ovase, 2001; Sokolova et al., 2009; Vukic et al., 2009 Westin et al., 2012; Kimura et al., 2018 Galimberti et al., 2006a; Zhang et al., 2013; Lee et al., 2018
CCL5 (RANTES)	CCR1 CCR3 CCR5	CCR5 deficient mice: ↑ Aβ	Lee et al., 2009	CCL5 ↑ in brain microvessels	Tripathy et al., 2010
CXCL8 (IL-8)	CXCR1 CXCR2	*In vitro* knock-down or pharmacological block of CXCR2: ↓ Aβ; activation of CXCR2: ↑ Aβ *In vivo* CXCR2 deficient mice: ↓ Aβ Recruitment of T-lymphocytes in the brain	Bakshi et al., 2008 Bakshi et al., 2011 Liu et al., 2010a	CXCL8 ↑ in brain CXCL8 ↑ in CSF CXCL8 ↑ in plasma CXCR2 ↑ in microglia and astrocytes CXCR2 ↑ in neuritic plaques	Ashutosh et al., 2011 Galimberti et al., 2006b Alsadany et al., 2013 Flynn et al., 2003 Xia et al., 1997
CXCL10 (IP-10)	CXCR3	CXCR3 deficiency mice: ↓ Aβ	Krauthausen et al., 2015	CXCL10 ↑ in brain	Xia et al., 2000
CXCL12 (SDF-1α)	CXCR4 CXCR7	*In vitro* CXCL12 prevents dendritic regression and neuronal apoptosis induced by Aβ	Raman et al., 2011	CXCL12 ↓ in plasma CXCL12 ↓ in brain CXCR4 ↑ in brain	Laske et al., 2008 Parachikova and Cotman, 2007 Parachikova and Cotman, 2007; Weeraratna et al., 2007
CX3CL1 (Fractalkine)	CX3CR1	CX3CR1 deficiency mice: ↑ p-*tau* CX3CR1 deficiency mice: ↓ Aβ	Bhaskar et al., 2010; Bolós et al., 2017; Nash et al., 2013 Lee et al., 2010; Liu et al., 2010b	CX3CL1 ↑ in plasma CX3CL1 ↑ in brain	Kim et al., 2008 Strobel et al., 2015
PROK2	PKR1 PKR2	*In vitro* Incubation of CNs with Aβ: ↑ PROK2/PKRs *In vivo* Non-transgenic AD mice model: ↑ PROK2/PKRs/TM4-7	Severini et al., 2015; Caioli et al., 2017 Severini et al., 2015; Lattanzi et al., 2019		

## Chemokines

Chemokines are chemotactic cytokines originally identified as factors regulating immune cell migration to sites of inflammation (Luster, 1998). This family, also widely expressed in the CNS, exerts its functions through chemokine receptors that belong to the superfamily of G-protein-coupled receptors. Most chemokines bind to more than one receptor, and several distinct chemokines share common receptor (Rossi and Zlotnik, 2000). Chemokines can be classified into sub-families on the basis of the sequential position of the first two of the four cysteine residues: CXC, CC, CX3C, and C (Bachelerie et al., 2013). The main alteration in chemokines and receptors discriminating pathophysiological inflammatory conditions from physiological ones is their increased expression as demonstrated in plasma, cerebrospinal fluid (CSF), and brain tissue of patients with AD. Microglia, astrocytes, and neurons are believed to be the main source of chemokines and their receptors’ production (Liu et al., 2014). In general, most of the chemokines and their receptors contribute to the neuroinflammatory component of AD by recruiting peripheral blood monocytes and promoting glial cell activation, even if emerging data hypothesize for some of them, a neuroprotective role.

**CCL2** or monocyte chemoattractant protein 1 (**MCP-1**), mostly produced by glial cells, seems to have a detrimental role in AD pathogenesis, as its overexpression has been found in brain (Sokolova et al., 2009; Vukic et al., 2009), mature senile plaques, microglia, and microvessels of AD patients (Grammas and Ovase, 2001). Clinical data of AD patients have shown an increase of CCL2 both in CSF and in plasma (Westin et al., 2012; Zhang et al., 2013), and, according to several authors, it correlates with the disease progression and the cognitive decline (Galimberti et al., 2006a; Kimura et al., 2018; Lee et al., 2018). Conversely, other studies report no association between CCL2 plasma levels and AD (Kim et al., 2011; Porcellini et al., 2013). On the other hand, the deficit of CCR2 (CCL2 receptor) may aggravate the disease progression. In transgenic mouse models of AD (Tg2576 mice and APPswe/PSEN1), Ccr2 deficiency accelerates memory deficits and disease progression increasing the Aβ soluble levels in the brain (El Khoury et al., 2007; Naert and Rivest, 2011). This could be due to an impaired macrophage recruitment, microglial accumulation, and Aβ clearance, which seems to be CCR2 dependent.

**CXCL8** (or **interleukin** 8) is produced in CNS by neurons, microglia, and astrocytes in response to proinflammatory signals. It has been found to be increased in serum, CSF, and brains of AD patients (Galimberti et al., 2006b; Ashutosh et al., 2011; Alsadany et al., 2013). Moreover, high levels of its receptors (CXCR2) have been reported in neuritic plaques of AD tissue, as well as in microglia and astrocytes (Xia et al., 1997; Flynn et al., 2003). Bakshi and collaborators have demonstrated *in vitro* that the knock-down or the pharmacological block of CXCR2 with the antagonist SB225002 induces an inhibition in Aβ release,through inhibition of γ-secretase, while the activation of CXCR2, with the exogenous chemokines hrIL8 and hrGRO-α, leads to an increase in Aβ. These data have been confirmed by the same authors in *in vivo* studies, in which Cxcr2 deficient mice show a reduction of Aβ that is associated to γ-secretase decrease (Bakshi et al., 2008, Bakshi et al., 2011). Furthermore, the intra-hippocampal Aβ_1–42 _injection induces microglial chemotactic response that involves the hippocampal overexpression of CXCL8/CXCR2 in a time-dependent manner (Ryu et al., 2015). The hippocampal Aβ_1–42 _injection also causes an up-regulation of CXCR2 in peripheral T cells associated with an increased T cell entry in the brain. These effects are reduced by intraperitoneal injection with the CXCR2 antagonist SB332235 (Liu et al., 2010a).

**CXCL10** (or **IP-10**). Clinical research in AD patients has demonstrated a positive correlation between the levels of CXCL10 in CSF and cognitive impairment (Galimberti et al., 2006b). CXCL10 is physiologically expressed in astrocytes and elevated in AD patients (Xia et al., 2000) and AD transgenic mice, where it co-localizes with Aβ plaques (Duan et al., 2008; Zaheer et al., 2013). CXCL10 binds to CXCR3 receptor that plays a critical role in the generation of AD pathology: in a transgenic AD mouse model, CXCR3 deficiency significantly reduces Aβ plaque formation and strongly diminishes Aβ peptide in brain tissue; this correlates with the improvement of the behavioral deficit (Krauthausen et al., 2015).

**CX3CL1** (also named **fractalkine**), unlike other chemokines, is produced as a transmembrane-anchored protein and exerts its functions as a membrane protein or as soluble isoforms. It is produced by neurons and astrocytes, whereas microglia constitutively express CX3CR1, its sole receptor (Mizuno et al., 2003). CX3CR1 is also partly expressed in astrocytes and neurons (Meucci et al., 2000; Cardona et al., 2006), suggesting that CX3CL1/CX3CR1 pathway plays a major role in neuron/microglia communication and allows neurons to regulate microglia activation (Limatola and Ransohoff, 2014). Clinical studies suggest that CX3CL1/CX3CR1 may participate to the development of AD pathogenesis: serum fractalkine is elevated in patients with mild AD, and its reduction is positively correlated with the cognitive decline (Kim et al., 2008). Moreover, an overexpression of CX3CL1 is present in the hippocampus of AD patients (Strobel et al., 2015). In AD animal models, different studies demonstrate a neuroprotective role of CX3CL1/CX3CR1 axis in *tau* pathology by favoring an anti-inflammatory context. Transgenic mice lacking CX3CR1 show increased *tau* phosphorylation and aggregation associated with microglial activation and behavioral impairments (Bhaskar et al., 2010; Bolós et al., 2017). In a mouse model of AD, Nash and collaborators have confirmed these results: the overexpression of soluble fractalkine reduces *tau* pathology with a significant reduction of microglial activation and neuronal loss (Nash et al., 2013). Regarding the CX3CL1/CX3CR1 in Aβ pathology, data seem to be divergent (Finneran and Nash, 2019). CX3CR1 deficiency in three different AD mouse models reduces β-amyloid deposition, enhancing Aβ phagocytic ability by microglia (Lee et al., 2010; Liu et al., 2010b). Similarly, upregulation of CX3CR1 in the hippocampus of rats injected with Aβ_1−40_, induces microglial activation, synaptic dysfunction, and cognitive impairments (Wu et al., 2013). On the other hand, CX3CR1 deficiency in mice overexpressing human amyloid precursor protein (APP) exhibits enhanced *tau* pathology, microglial activation, expression of proinflammatory markers (IL-1β, TNF-α, and IL-6), neuronal death in the dentate gyrus, and worsened learning abilities (Cho et al., 2011). Moreover, in the same mouse model, a decrease in CX3CL1 has been shown in cerebral cortex and hippocampus (Duan et al., 2008).

**CCL5** (also known as **RANTES**) and its receptors CCR5 are found on endothelial cells, glia, and neurons throughout the brain. The functional role of RANTES in AD is not completely clear. Indeed, different *in vitro* studies have shown that treatment of neuronal cultures with RANTES enhances neuronal survival (Tripathy et al., 2010) and protects against Aβ toxicity (Bruno et al., 2000). Conversely, *in vivo* studies have reported that RANTES and CCR5 are increased in transgenic mice brain (Subramanian et al., 2010; Haskins et al., 2016) as well as in microvessels of AD human brain (Tripathy et al., 2010). CCR5 also binds other chemokines such as CCL3, whose role in AD is still not clear. However, mice lacking CCL3 or CCR5 exhibit a reduced glial activation and an improvement of spatial learning deficit induced by the intracerebroventricular (ICV) Aβ_1−40_ injection (Passos et al., 2009). Moreover, the ICV administration of CCL3 in mice impairs synaptic transmission and spatial memory; these effects are reverted by a CCR5 antagonist (Maraviroc) (Marciniak et al., 2015). Furthermore, CCR5 deficiency results in enhanced long-term potentiation (LTP) and learning/memory performances, while neuronal CCR5 overexpression causes memory deficits (Zhou et al., 2016). In line with these findings, Lee and co-authors have demonstrated that CCR5 deficient mice have higher accumulation of Aβ, associated with astrocyte activation in the brain, and an impairment of memory and learning functions (Lee et al., 2009).

The chemokine **CXCL12** (**SDF-1α**), and its receptor CXCR4, are expressed and widely detected in the developing and adult CNS (Ma et al., 1998; Zou et al., 1998; Banisadr et al., 2002; Schönemeier et al., 2008). In addition to their role in neuroinflammation, they regulate neuronal excitability and synaptic transmission (Limatola et al., 2000) and modulate neuronal firing and neuron/glia communication (Bezzi et al., 2001). An upregulation of Cxcr4 gene and protein levels has been found in the brains of AD (Weeraratna et al., 2007). Furthermore, a decrease in plasma CXCL12 is reported in early AD patients and negatively correlated with CSF *tau* protein levels (Laske et al., 2008). In agreement with these results, Parachikova and Cotman (2007) show an increase of CXCR4 protein expression and a decrease of CXCL12 in the hippocampus of AD patients. However, the authors also demonstrate that transgenic AD mice, in which the mutation induces an overproduction of human APP, exhibit a reduction of CXCL12 and CXCR4 that correlates with deficits in different cognitive tasks. Moreover, chronic administration of a CXCR4 antagonist (AMD3100) results in impaired learning and memory in young non-transgenic mice, thus supporting the hypothesis that low levels of CXCL12/CXCR4 are linked to cognitive deficits (Lu et al., 2002). It has also been demonstrated that pre-treatment with CXCL12 inhibits the deleterious effect induced by the ICV injections of Aβ, suggesting that the beneficial effect of CXCL12/CXCR4 on memory and learning in AD could be linked to the prevention of dendritic regression and neuronal apoptosis induced by Aβ (Raman et al., 2011). In agreement with these data, a recent study demonstrates that CXCL12 mediates the neuroprotective and anti-amyloidogenic actions of human painless NGF (hNGFp) treatment in 5xFAD mice, transgenic mice that co-overexpress five familial AD mutant forms of human APP and presenilin 1 (Capsoni et al., 2017). Noteworthy, CXCL12 also binds CXCR7 that seems to be involved in the progression of various CNS pathologies including AD (Puchert et al., 2017).

## Prokineticins

The amphibian Bv8 and mammalian prokineticin 2 (PROK2) are two secreted bioactive peptides that belong to the prokineticin (PK) family. Prokineticins are highly conserved across the species. They are characterized by the presence of a conserved N-terminal sequence AVITGA, 10 cysteine residues, that create five disulphide-bridged motifs and a tryptophan residue in position 24. A degree of similarity between prokineticin and defensins, a subclass of cationic antimicrobial peptides involved in innate immunity that, similarly to prokineticins, contain a high number of cysteine residues, was reported. However, their small size (8 kDa), signaling mechanisms, receptor coupling (G protein-coupled receptors, GPCR), as well as chemotactic and immune-modulatory functions classify prokineticins as chemokines (Monnier and Samson, 2008; see also Negri and Ferrara, 2018).

Prokineticins activate two GPCR, prokineticin receptor 1 (PKR1) and 2 (PKR2), widely distributed in different organs and tissues as well as in CNS, in which PKR2 results more expressed. Both receptors are highly conserved sharing 85% amino acid identity and diverging mainly in their N-terminal region (Kaser et al., 2003). The PKRs are coupled to Gq, Gi, and Gs depending on the type of cellular localization and activate different intracellular signal pathways (for an explicative review, see Negri and Ferrara, 2018). It has also been reported that PKR2, at least in human neutrophils, undergoes dimerization (Marsango et al., 2011). Furthermore, in saccharomyces, the dimerization takes place from interactions between transmembrane domains TMs 4 and 5, with a specific role playing by TM5 on PKR2 function (Sposini et al., 2015).

PROK2 is abundantly expressed in CNS and associated with multiple physiological and pathological functions such as circadian rhythm, neurogenesis, angiogenesis, pain, inflammation, and neuroinflammation (Cheng et al., 2006; Zinni et al., 2017; Negri and Ferrara, 2018; Negri and Maftei, 2018).

The first evidence suggesting the potential role of PROK2/PKRs in AD comes from *in vitro* and *in vivo* studies demonstrating their overexpression following Aβ_1–42_ exposure. The incubation of primary cortical cell cultures (CNs) with Aβ_1–42_ increases the PROK2/PKR mRNA and protein expression in a time-dependent way with a maximum increase at 48 h.Immunofluorescence studies revealed that PKR1 only increases in neuronal cell body, while PROK2 and PKR2 increase both in neurons and in astrocytes (Severini et al., 2015; Caioli et al., 2017). The Aβ_1–42_-induced PROK2 overexpression is strongly reduced by preincubation with PC1, a non-peptide PKR antagonist (Balboni et al., 2008; Congiu et al., 2014). Interestingly, PC1 reverts the Aβ_1–42_-induced neuronal death (in a concentration-dependent way), suggesting that reducing the activation of the PK system could be beneficial against Aβ_1–42_–induced neuronal toxicity.In support of these data, we found that the incubation of CNs with Bv8, the amphibian homologue of PROK2, induces neuronal apoptosis, comparable to the one exerted by Aβ peptide. It is also noteworthy that Bv8-induced neurotoxic effects are concentration-dependent, being achieved specifically at picomolar (and not at nanomolar) concentrations, and are blocked by pre-incubation with PC1. These low concentrations of Bv8, which induce the harmful effects, could be correlated with the small amount of PROK2, eventually released during Aβ incubation. Along this line, a harmful effect of PROK2 at picomolar concentration is reported in cerebral ischemia (Cheng et al., 2012).

It is known that Aβ_1–42 _induces a significant increase of the ionic current through the AMPA receptors (Parameshwaran et al., 2008; Wang et al., 2010). We have demonstrated that in neurons from CNs, Aβ treatment affects the glutamatergic transmission through the involvement of the PK system (Caioli et al., 2017). Indeed, CNs incubated with Bv8, as well as with Aβ_1–42_, exhibit an alteration of glutamatergic transmission that results in increase in AMPA receptor ionic currents, measured both as evoked and as spontaneous, which are blocked by PC1. The up-modulation of AMPA ionic currents is not due to modifications of the GluR1 or GluR2 AMPA receptor subunit expression; rather, it appears to be mediated by modification in phosphorylation of AMPA subunits by the activation of protein kinase C (PKC) intracellular pathway. This is confirmed by the antagonist effect of PKC inhibitor (Go6983). Of note, PC1 also reduces neuronal death induced by kainite, thus exerting a protective action.

The involvement of the PK system in AD is also strongly supported by *in vivo* evidences. In the Tg2576 (TG) transgenic mouse model, PC1 exposure prevents LTP impairment in hippocampal slices, indicating that the pharmacological block of PKRs in TG neurons is sufficient to rescue the synaptic plasticity and to protect against the deleterious effect of PROK2. No changes are observed in age-matched wild-type controls, suggesting that PKR blockade does not affect synaptic plasticity in physiological conditions. In a non-transgenic animal model of AD, induced by ICV injection of Aβ_1–42_, PROK2 and PKR mRNA expression is increased in both cortex and hippocampus (Severini et al., 2015). In addition, PKR2 mRNA levels in hippocampus are found to be increased not only in the early times (24 h after Aβ_1–42_ injection) but also in the later stage (14 days after Aβ_1–42_ injection), indicating a significant role of PK system in the progression of pathology (Lattanzi et al., 2019).

In AD, the differential expression and the alternative splicing of genes notoriously involved in the pathology, such as APOE, APP, or *tau*, may contribute to the pathogenesis of the disease (Love et al., 2015). Alternative splicing of GPCR is a common mechanism that allows the formation of cell-specific isoforms with different biological activity in health and disease (Einstein et al., 2008; Wang et al., 2008).

**Figure 1:**
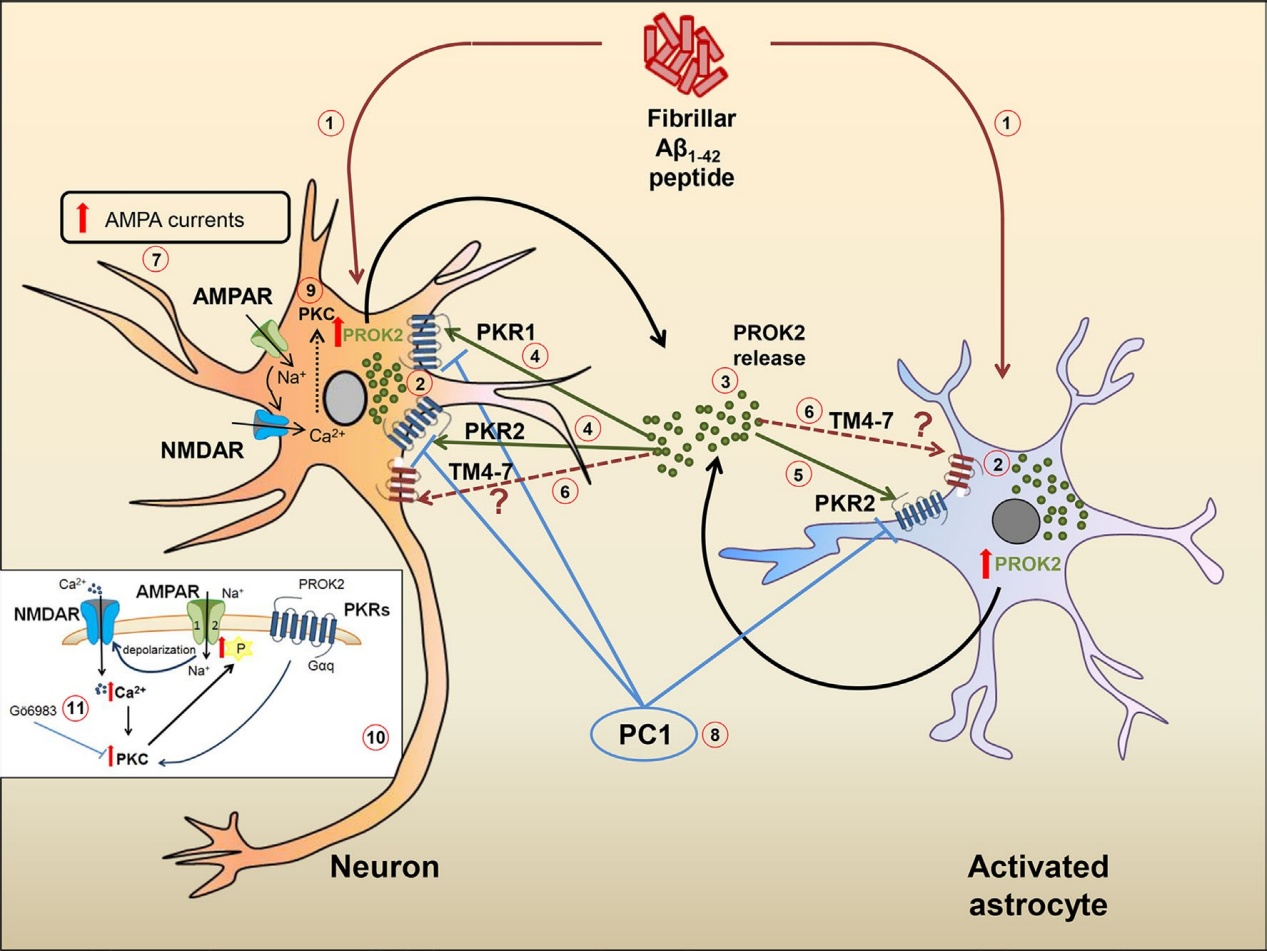
Hypothetical role of the PK system in Aβ-mediated cell toxicity. Ab_1−42 _peptide **(1)** induces an increase of PROK2 in both neurons and astrocytes **(2)**. Once released **(3)**, PROK2 binds to PKR1 and PKR2 on neurons **(4)** and on PKR2 localized on astrocytes **(5)**. PROK2 may also bind to the PKR2-truncated isoform (TM4-7) whose cell type localization and function is still unknown **(6)**. At neuronal level, the increase of AMPA-receptor ionic currents induced by Aβ (but also by Bv8) **(7)** seems to be mediated by the PK system, as it is blocked by the PKR antagonist PC1 **(8)**. The up-modulation of AMPA ionic currents may be mediated by the activation of PKC intracellular pathways **(9)** [in detail illustrated in the small box **(10)**], as confirmed by the antagonistic effect of PKC inhibitor Go6983 **(11)**.

In a recent paper, a PKR2 splice variant has been identified in rat hippocampus and called TM4-7 since the lack of the second exon gives rise to a receptor containing only four transmembrane domains. Interestingly, in the preclinical model of AD induced by ICV injection of Aβ in rat, the expression of TM4-7 receptor isoform is strongly up-regulated in the hippocampus and the expression ratio between the sliced form TM4-7 and the long form PKR2 increases with the progression of the disease, reaching maximum levels 35 days after Aβ injection (Lattanzi et al., 2019). We can hypothesize that, as already observed in yeast (Lattanzi et al., 2019), also in the brain, TM4–7 may generate homodimer or heterodimer functional receptors with PKR2, so adding versatility and complexity to the already complex mechanisms of brain regulation induced by PROK2 and its receptors. A summary of the hypothetical role of PK system in Aβ-induced cell toxicity is shown in **Figure 1**.

## Conclusions

The work summarized in the present review indicates that chemokines and their receptors are crucial neuroinflammatory actors for AD pathogenesis and/or progression. However, many gaps remain in the knowledge on the specific role of neuroinflammation in AD, which impede the development of successful therapeutic strategies. Prokineticins represent a new class of chemokine-like proteins involved in Aβ-induced toxicity and represent an innovative approach for the study of AD pathogenesis. Although in our laboratory many studies are still in progress, the success of the pharmacological blockade of PKRs (that reduces the Aβ-induced neuronal death) leads us to hope for a future promising pharmacotherapeutic strategy of AD. This is particularly relevant considering that, to date, there are no drugs that block or slow down the progression of the disease.

## Author Contributions

ARZ and DM made literature search and wrote the first draft of the manuscript. PC and RL designed the aim of the review. DM drew the figure. All authors contributed to reading and approving the final version of the manuscript.

## Conflict of Interest Statement

The authors declare that the research was conducted in the absence of any commercial or financial relationships that could be construed as a potential conflict of interest.
